# Human cardiac fibroblasts produce pro-inflammatory cytokines upon TLRs and RLRs stimulation

**DOI:** 10.1007/s11010-021-04157-7

**Published:** 2021-04-21

**Authors:** Zhe Li, Tuan T. Nguyen, Alan Valaperti

**Affiliations:** Department of Immunology, University Hospital Zurich, Gloriastrasse 23, CH-8091 Zurich, Switzerland

**Keywords:** Cytokines, Human Cardiac Fibroblasts, Inflammation, Interleukins

## Abstract

Heart inflammation is one of the major causes of heart damage that leads to dilated cardiomyopathy and often progresses to end-stage heart failure. In the present study, we aimed to assess whether human cardiac cells could release immune mediators upon stimulation of Toll-like receptors (TLRs) and Retinoic acid-inducible gene (RIG)-I-like receptors (RLRs).

Commercially available human cardiac fibroblasts and an immortalized human cardiomyocyte cell line were stimulated in vitro with TLR2, TLR3, and TLR4 agonists. In addition, cytosolic RLRs were activated in cardiac cells after transfection of polyinosinic-polycytidylic acid (PolyIC). Upon stimulation of TLR3, TLR4, MDA5, and RIG-I, but not upon stimulation of TLR2, human cardiac fibroblasts produced high amounts of the pro-inflammatory cytokines IL-6 and IL-8. On the contrary, the immortalized human cardiomyocyte cell line was unresponsive to the tested TLRs agonists. Upon RLRs stimulation, cardiac fibroblasts, and to a lesser extent the cardiomyocyte cell line, induced anti-viral IFN-β expression.

These data demonstrate that human cardiac fibroblasts and an immortalized human cardiomyocyte cell line differently respond to various TLRs and RLRs ligands. In particular, human cardiac fibroblasts were able to induce pro-inflammatory and anti-viral cytokines on their own. These aspects will contribute to better understand the immunological function of the different cell populations that make up the cardiac tissue.

## INTRODUCTION

Inflammation of the heart muscle, also called myocarditis, can result from infections, autoimmune reactions targeting the heart tissue, or toxic compounds that directly damage the heart [1]. Without specific supportive therapy, heart inflammation leads to dilated cardiomyopathy and often progresses to end-stage heart failure [2]. Several immune cells reside in the heart tissue and play a protective role after cardiac injury or against invading cardiotropic viruses [3]. In addition, progenitor cells migrate from the bone marrow and infiltrate into the inflamed heart [4]. The majority of these cells are mononuclear phagocytes, such as monocytes and macrophages [5–7]. Their various functions span from promoting heart inflammation and fibrosis to supporting heart repair after cardiac injury and reduction of inflammation and infection [4, 6, 8, 9]. Activation of monocytes and macrophages is triggered by various damage-associated molecular patterns (DAMPs), which target specific pattern recognition receptors (PRR), such as Toll-like receptors (TLRs) and Retinoic acid-inducible gene (RIG)-I-like receptors (RLRs) on the surface, in the endosomes or in the cytosol of these cells [10]. The resulting immune response leads to NF-kB-dependent transcription of pro-inflammatory cytokines [11, 12].

In the last few years some interesting studies demonstrated that also cardiac cells of mouse and human origin express TLRs [13]. Among TLRs, TLR2 recognizes triacyl and diacyl lipopeptide of Gram-positive bacteria together with TLR1 and TLR6 [14], while TLR3 specifically recognizes viral double-stranded (ds)RNA in the endosomes and promotes type I interferons (IFNs) responses [15]. Lipopolysaccharides (LPS) activate TLR4 [16]. Upon stimulation of all TLRs, except for TLR3, the adapter protein MyD88 promotes phosphorylation of the kinases IRAK1/2/4, which then dissociate from MyD88 and associate with the ubiquitin ligase TRAF6 to ultimately promote the phosphorylation of the transcription factor NF-kB [17]. The classical NF-kB pathway regulates the expression of pro-inflammatory cytokines, such as *IL-6, TNF-α*, and *IP-10* [18].

TLRs play a key role in the pathogenesis of cardiac conditions, such as in myocardial infarction (MI) and myocarditis [3, 19]. Among the ten known TLRs, a large amount of previous studies investigated the function of TLR2, TLR3, and TLR4 in heart inflammation. TLR2 has been shown to modulate T helper (Th) cell subpopulations in viral myocarditis [20], while in mice with induced ischemia/reperfusion (I/R) injury, TLR2 facilitates the recruitment of inflammatory lymphocytes to the damaged heart [21]. The activation of TLR3, which has been evaluated in anti-viral responses induced after CVB3 or CVB4 infection in the mouse model of viral myocarditis, exerts a protective anti-viral function in these mice, reducing viral load in heart tissue by promoting type I and type II IFN production [22, 23]. TLR4, which worsens the outcome of mice with I/R injury and myocarditis, promotes pro-inflammatory immune responses by enhancing the production of pro-inflammatory cytokines [13, 24, 25].

The RNA expression of several TLRs has been reported in mouse heart tissue, and after myocardial injury or myocarditis, their expression was further increased [25–27]. In the murine cardiomyocytes (CM) cell line HL-1 and in primary mouse cardiac fibroblasts (CF), after LPS stimulation, IL-6, TNF-α, CXCL10, and CXCL1 are highly increased [27, 28], while rat CM secrete increased amounts of TNF-α [29]. TLR2 stimulation in primary mouse CM leads to elevated production of TNF-α and IFN-γ [30]. In total human cardiac tissue, the RNA expression of all 10 TLRs has been described, but the highest relative levels measured are for TLR4, TLR2 and TLR3 [31]. After in silico protein expression analysis, it has been extrapolated that TLR4 and TLR9 show high proteins expression in healthy human heart tissue [26].

Besides TLRs, cytosolic RIG-I-like receptors (RLRs) agonists induce important immunological responses [32]. The RLRs include three members, namely MDA5, RIG-I, and LGP2 [33] and their signalling pathways induce phosphorylation of NF-kB and interferon regulatory factor (IRF)3 [34]. The importance of MDA5 in promoting anti-viral type I interferon (IFN) responses has been described in a mouse model of Coxsackievirus B3 (CVB3)-induced myocarditis [6, 35]. In addition, infection of mouse cardiac cells and other human cell lines with CVB3 in vitro demonstrate the relevance of MDA5 in supporting efficient type I IFN-dependent anti-viral responses [6, 36].

In mouse primary cardiac cells, nuclear translocation and phosphorylation of NF-kB is cell-type specific [37]. TLRs and RLRs, by recognizing various microbial components, mount an important innate immune defense for the host. However, extended and inappropriate host immune responses can lead to heart damage and eventually to adverse clinical outcomes. It is not yet known how human heart cells react to inflammatory stimuli and if the NF-kB activation may be cell-type specific among human heart cells. In this study we investigated if human CF and an immortalized human cardiomyocyte cell line express pro-inflammatory cytokines and type I IFN upon TLRs and RLRs stimulation in vitro.

## MATERIALS AND METHODS

### Cell Culture

Human cardiac fibroblasts (CF) were purchased from Sigma-Aldrich (Cat. No. 306–05). These human CF were isolated from the ventricles of adult human heart. Human CF were cultivated in T-75 flasks with 20 ml Cardiac Fibroblast Growth Medium (Sigma-Aldrich).

The immortalized AC16 human cardiomyocyte cell line (IHCM) was purchased from EMD Millipore (Cat. No. SCC109). This is a proliferating human cardiomyocyte cell line derived from the fusion of primary cells from adult human ventricular heart tissues with transformed SV40. The AC16 cardiomyocytes have retained the nuclear DNA and the mitochondrial DNA of the primary cardiomyocytes. AC16 human cardiomyocytes were cultivated in T-75 flasks with 20 ml DMEM/Nutrient Mixture F-12 Ham (Sigma-Aldrich) containing 2 mM L-Glutamine (EMD Millipore), 12.5% FBS (EMD Millipore) and 1X Penicillin–Streptomycin. Experiments were performed with cells on passage 2 and passage 3. After passage 3, cells were wasted. For further experiments, new aliquots were thawed and cultivated only up to passage 3.

### Cell Stimulation

Cell stimulation was performed in 24-well plates. 5 × 10e4 cells were stimulated with Pam3CSK4 (synthetic triacylated lipoprotein), PolyIC (polyinosinic-polycytidylic acid), or LPS (all purchased from InvivoGen) for 2, 4, 8, and 24 h. For intracellular stimulation, cardiac cells were plated 24 h before stimulation to reach 80% confluence. Transfection of PolyIC was performed with the LipoD293 In Vitro DNA Transfection Reagent (SignaGen) in DMEM with high glucose, as previously shown [35]. Transfection efficiency was checked after 24 h.

### Small Interfering (si)RNA Transfection

For knockdown experiments, TLR3, MDA5, RIG-I, and Mock siRNAs were purchased from Qiagen. Cells were cultured to 80% confluency in 24-well plates and transfected with 80 pmol siRNA and GenMute siRNA Transfection Reagent (SignaGen), according to the manufacturer’s protocol. Twenty-four hours after siRNA transfection, cells were stimulated with specific TLRs and RLRs agonists.

### Quantitative RT-PCR (qRT-PCR)

To measure cytokine expression at the RNA level, RNA was isolated with TRI Reagent (Sigma-Aldrich) according to the manufacturer’s protocol. The concentration of the extracted RNA was determined with a NanoDrop spectrophotometer (Thermo Scientific) and the A260/A280 ratio was always ≥ 1.8. Reverse transcription was performed with 1 µg RNA and the High-Capacity cDNA Reverse Transcription Kit (Applied Biosystems). The KAPA SYBR FAST qPCR Master Mix (2X) Kit (Sigma-Aldrich) was used to quantify gene expression on a 7900-HT Fast Real Time PCR instrument (Applied Biosystems). The 2^−ΔΔCt^ method was used for qRT-PCR gene expression analysis [38]. Genes of interest were compared with the housekeeping gene *GAPDH*. Primers used to measure the RNA expression of *IL-1β, IL-6, IL-8, IFN-β, TNF-β, TLR2, TLR3, and TLR4* were: *IL-1β* forward: CTGTCCTGCGTGTTGAAAGA; *IL-1β* reverse: GGGAACTGGGCAGACTCAAA. *IL-6* forward: GGAGACTTGCCTGGTGAAAA; *IL-6* reverse: GTCAGGGGTGGTTATTGCAT. *IL-8* forward: ACTGAGAGTGATTGAGAGTGGAC; *IL-8* reverse: AACCCTCTGCACCCAGTTTTC. *TNF-α* forward: CCCCAGGGACCTCTCTCTAATC; *TNF-α* reverse: GGTTTGCTACAACATGGGCTACA. *IFN-β* forward: CAGCAATTTTCAGTGTCAGAAGC; *IFN-β* reverse: TCATCCTGTCCTTGAGGCAGT. *TLR2* forward: CTTCACTCAGGAGCAGCAAGCA; *TLR2* reverse: ACACCAGTGCTGTCCTGTGACA. *TLR3* forward: GCGCTAAAAAGTGAAGAACTGGAT; *TLR3* reverse: GCTGGACATTGTTCAGAAAGAGG. *TLR4* forward: CCCTGAGGCATTTAGGCAGCTA; *TLR4* reverse: AGGTAGAGAGGTGGCTTAGGCT.

### Cytokine Measurement in Supernatants

Cytokine concentrations in cell-free supernatants were measured on a MagPix (Luminex Corporation) with the magnetic Human High Sensitivity Cytokine Base Kit A, which included IL-1β, IL-6, IL-8, and TNF-α (R&D Systems Bio-Techne). Data were analyzed with the xPONENT software. Supernatants were collected 24 h after stimulation with different concentrations of TLRs agonists.

### Measurement of TLR3 by Flow Cytometry (FACS)

Human cardiac fibroblasts were stained using fluorochrome-conjugated human-specific antibodies against TLR3 (purchased from BioLegend). Samples were acquired with a FACSCanto II cell analyzer (BD Biosciencs) and analyzed using the FlowJo (Tree Star) software. Intracellular staining was performed with the Intracellular Fixation and Permeabilization Buffer Set (eBioscience), which was used according to the manufacturer’s instructions. Data were collected from a live gate using forward/side scatter plot.

### Statistics

Cytokines expressed at the RNA level were plotted over time and their significance was tested by repeated measured analysis of variance (two-way ANOVA) followed by Bonferroni post hoc testing. Cytokine concentrations in pg/ml between two groups were tested with the unpaired two-tailed Student t test and with the two-way ANOVA with Bonferroni post hoc testing when three or more groups were compared. Statistical analysis was conducted using the Prism 6 software (GraphPad Software). All data were expressed as mean ± s.d. Differences were considered statistically significant for *p* < 0.05.

## RESULTS

### Expression of TLRs on Human Cardiac Fibroblasts (CF) and on the Immortalized AC16 Human Cardiomyocyte Cell Line (IHCM)

**Fig. 1:**
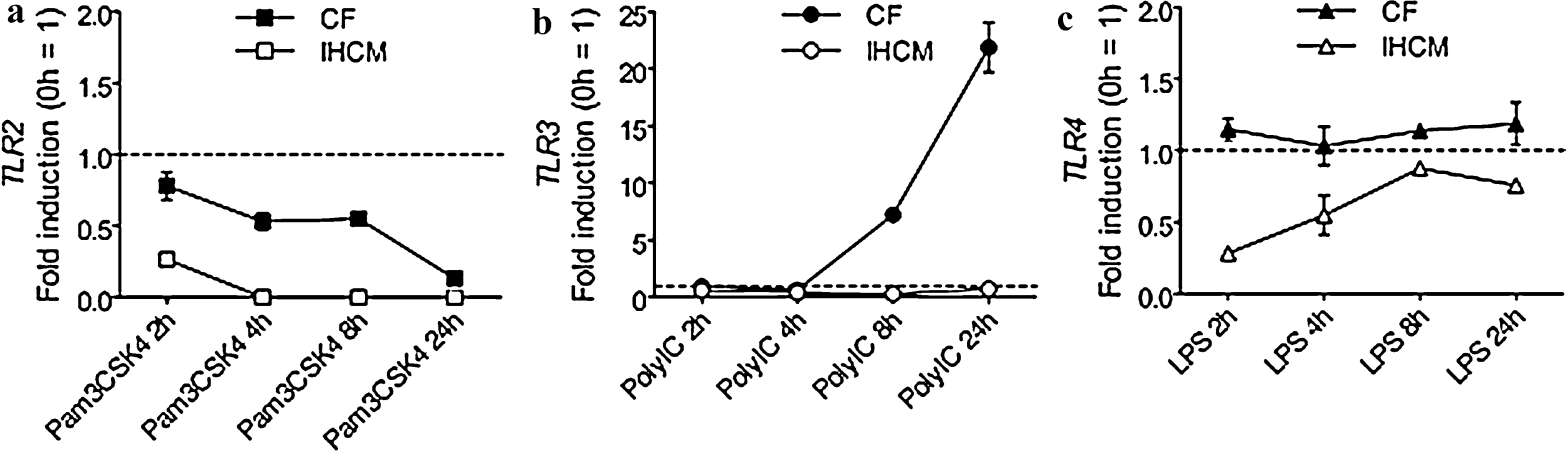
Upon TLRs stimulation, human CF show only *TLR3* expression, while the IHCM does not express *TLR2*, *TLR3*, and *TLR4*. (**a**-**c**) Human CF and IHCM were stimulated for 2, 4, 8, and 24 h with 10 ng/ml Pam3CSK4 (**a**), with 10 ng/ml PolyIC (**b**), or with 20 ng/ml LPS (**c**). Transcription of *TLR2*, *TLR3*, and *TLR4* was determined by real-time RT-PCR. Dashed line set at “1” represents unstimulated cells. Means ± s.d. and values measured from one out of three independent experiments performed in duplicates are shown

A previous study showed that in total human heart tissue, among the 10 tested TLRs, the highest expression was measured for TLR2, TLR3, and TLR4 [31]. Before starting to investigate how these three TLRs could modulate the expression and production of pro-inflammatory cytokines in human CF and in the IHCM, their expression had to be verified in human CF and in the IHCM. Interestingly, neither CF nor the IHCM expressed TLR2, while the expression of TLR4 was slightly increased after 8 and 24 h stimulation with LPS in CF but not in the IHCM, and TLR3 was highly expressed after 8 and 24 h stimulation with PolyIC in CF but not in the IHCM (Fig. 1a, b, and c). These results suggest that CF may play a relevant role in inducing PolyIC- and LPS-dependent inflammatory responses, while TLR2 is irrelevant or has just a marginal function in cardiac cell-mediated pro-inflammatory cytokine production.

### Cytokine Expression in Human CF and in the IHCM upon TLR2 and TLR4 Stimulation

**Fig. 2:**
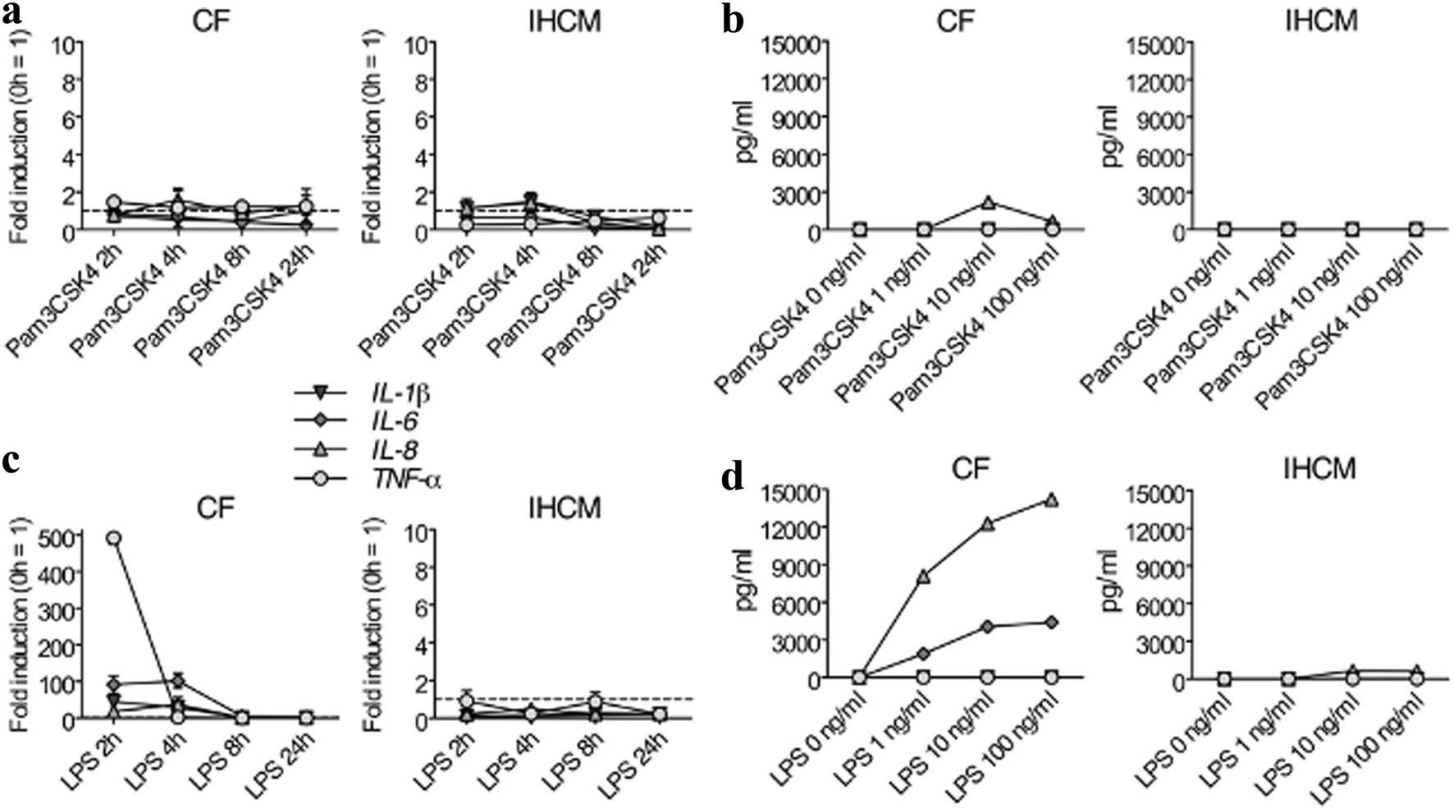
After TLR4 stimulation, human CF, but not the IHCM, induce pro-inflammatory cytokine production, while after TLR2 stimulation, both human CF and the IHCM are mostly unresponsive. (**a**) Human CF and IHCM were stimulated for 2, 4, 8, and 24 h with 10 ng/ml Pam3CSK4. Transcription of *IL-1β*, *IL-6*, *IL-8*, and *TNF-α*, determined by real-time RT-PCR, was almost absent. (**b**) Human CF and IHCM were stimulated for 24 h with different concentrations of Pam3CSK4 (1, 10, and 100 ng/ml) or left untreated (Pam3CSK4 0 ng/ml). IL-1β, IL-6, IL-8, and TNF-α, measured at the protein level in cell-free supernatants, were mostly negative. (**c**) Human CF and IHCM were stimulated for 2, 4, 8, and 24 h with 10 ng/ml LPS. Transcription of *IL-1β*, *IL-6*, *IL-8*, and *TNF-α*, determined by real-time RT-PCR, was only measurable in human CF, while in the IHCM, transcription was negative. (**d**) Human CF and IHCM were stimulated for 24 h with different concentrations of LPS (1, 10, and 100 ng/ml) or left untreated (LPS 0 ng/ml). IL-6, IL-8, but not IL-1β and TNF-α, which were all measured at the protein level in cell-free supernatants, were positive in human CF, while in the IHCM, all cytokines were negative. Dashed line set at “1” in (a) and (c) represents unstimulated cells. Means ± s.d. and values measured form one out of two independent experiments performed in duplicates are shown

Most relevant cytokines that induce acute phase proteins under control of NF-kB are IL-1β, IL-6, IL-8, and TNF-α [39]. Differences in NF-kB activation have been observed between CF and CM in rodents [37, 40]. Therefore, since cytokines are involved in heart inflammation [3], we aimed to clarify if human CF and the IHCM differently express inflammatory cytokines after TLRs stimulation. Since TLR2 was not expressed neither in CF nor in IHCM (Fig. 1a), it was not surprising that stimulation with Pam3CSK4 did not induce any cytokine expression at the RNA level in CF and in the IHCM, while just a slightly increase of IL-8 in CF at the protein level was observed (Fig. 2a and b). After stimulation with LPS, strong initial *TNF-α*, *IL-6*, and *IL-8* RNA expression was measured in CF, while the IHCM was fully unresponsive (Fig. 2c). The protein levels, however, did not fully mirrored RNA expression. Indeed, TNF-α, as well as IL-1β, were absent in supernatants of LPS-stimulated CF, while IL-6 and IL-8 were measured at high levels, which rose in parallel with the increased concentration of LPS (Fig. 2d). In supernatants of LPS-stimulated IHCM, just IL-8 was slightly expressed when LPS was used at high concentrations, while no other cytokines were detectable (Fig. 2d). These data indicate that CF can be induced to produce IL-6 and IL-8.

### TLR3 Regulates Pro-Inflammatory Cytokines in PolyIC-Stimulated Human CF

**Fig. 3:**
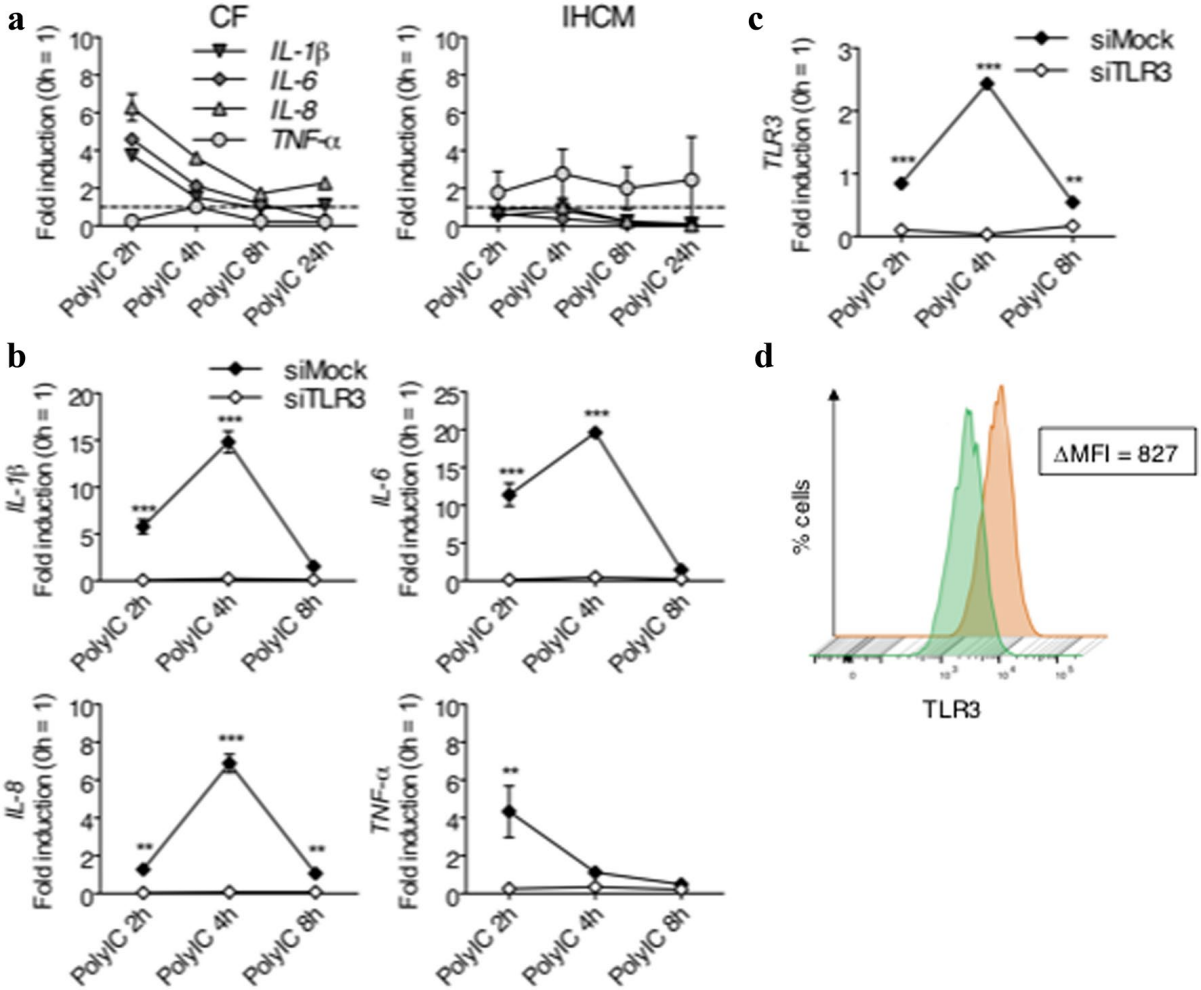
Production of pro-inflammatory cytokines in human CF is TLR3-dependent. (**a**) Human CF and IHCM were stimulated for 2, 4, 8, and 24 h with 10 ng/ml PolyIC. Transcription of *IL-1β*, *IL-6*, and *IL-8*, determined by real-time RT-PCR, was positive in human CF, while in the IHCM, only *TNF-α* was positive. (**b**-**c**) Knockdown of TLR3 with siRNA in human CF stimulated for 2, 4, and 8 h with 10 ng/ml PolyIC. SiMock was used as a control. Real-time RT-PCR used to measure transcription of *IL-1β*, *IL-6*, *IL-8*, *TNF-α* demonstrated that in the absence of TLR3, human CF did not express pro-inflammatory cytokines upon PolyIC stimulation (**b**). *TLR3* knock-down efficiency was also determined by real-time RT-PCR (**c**). (**d**) Knockdown of TLR3 with siRNA in human CF stimulated for 8 h with 10 ng/ml PolyIC (green histogram). SiMock was used as a control (orange histogram). FACS analysis was performed for human CF stained with PE-conjugated anti-TLR3 antibodies. The difference between the mean fluorescence intensity (MFI) of the human CF knocked down with siTLR3 and the MFI of the siMock control human CF was 827 MFI. Means ± s.d. and values measured form one out of two independent experiments performed in duplicates are shown. Two-way ANOVA with Bonferroni post hoc testing: **p < 0.01, ***p < 0.001 for siMock vs. siTLR3

PolyIC is the agonist of TLR3 in the endosome [41]. It is not known how different human cardiac cells react to PolyIC stimulation, therefore *IL-1β*, *IL-6*, *IL-8*, and *TNF-α* were measured at the RNA level after PolyIC stimulation. In CF, *IL-1β*, *IL-6*, and *IL-8* expression was increased, while *TNF-α* did not show any variation compared to the baseline value (Fig. 3a). On the contrary, in the IHCM, only *TNF-α* was increased, while all other cytokines were comparable to the baseline values (Fig. 3a).

To further understand the role of TLR3 in cardiac cells, TLR3 expression on CF was knocked down with specific human TLR3 siRNA (siTLR3). Mock siRNA (siMock) was used as a control. After stimulation with 10 ng/ml PolyIC, the expression of all cytokines was abrogated in siTLR3-transfected CF, while siMock-transfected CF still showed normal cytokine expression (Fig. 3b). Reduction of TLR3 observed in siTLR3-transfected CF at the RNA level measured by quantitative PCR (Fig. 3c), as well as at the protein level determined by FACS (Fig. 3d), demonstrated the high efficiency of the siRNA used in these experiments. Taking together, TLR3 contributes to pro-inflammatory responses in human cardiac cells.

### MDA5 and RIG-I Contribute to Pro-inflammatory Cytokine Expression in Human CF after PolyIC Stimulation in the Cytosol

**Fig. 4:**
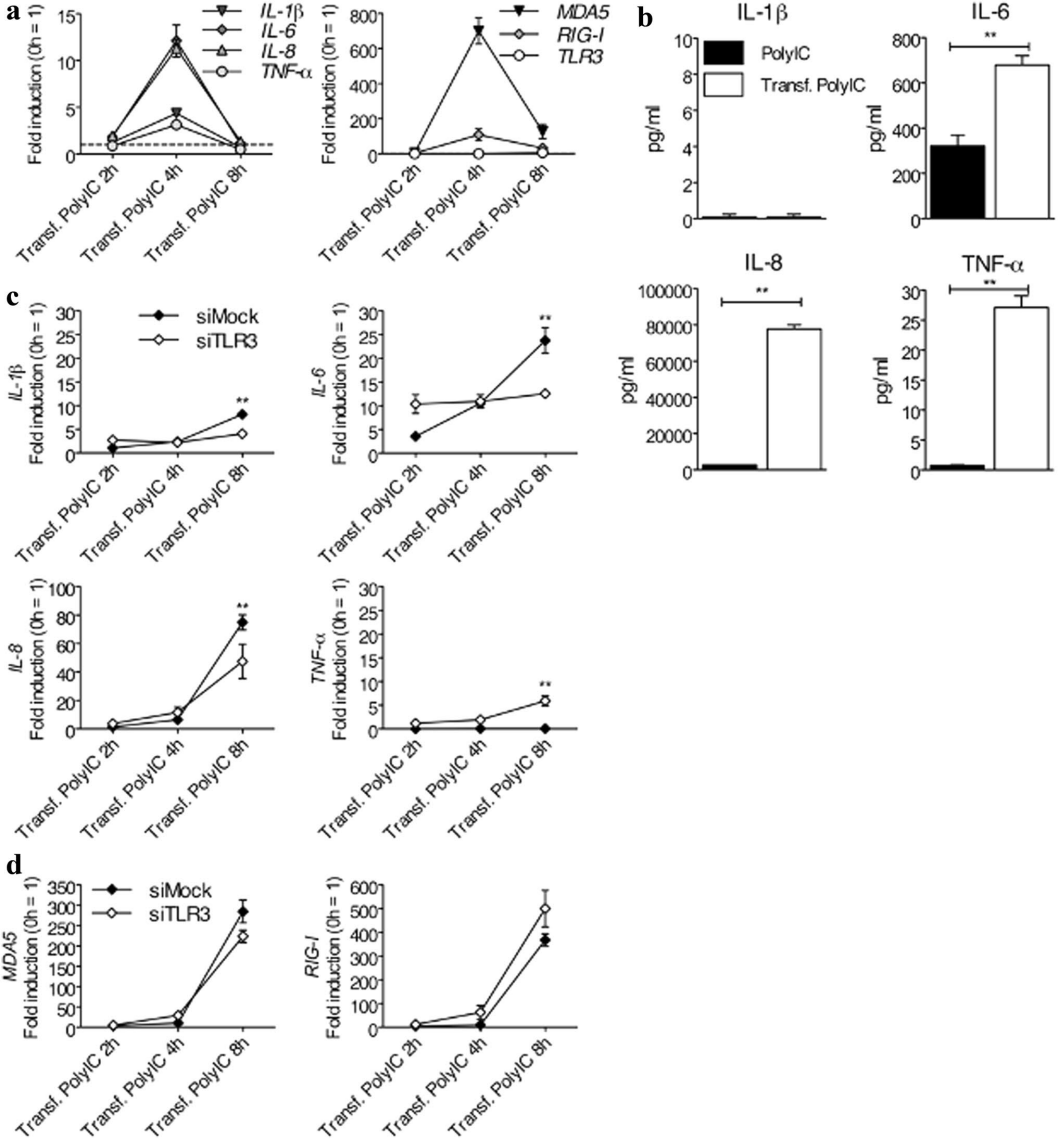
Human CF stimulated with transfected PolyIC produce higher levels of pro-inflammatory cytokines than after stimulation with untransfected PolyIC. (**a**) Human CF were stimulated for 2, 4, and 8 h with 1 ng/ml of intracellularly transfected PolyIC. Transcription of *IL-1β*, *IL-6*, *IL-8*, and *TNF-α* (left panel) and transcription of *MDA5* and *RIG-I*, but not of *TLR3* (right panel), all determined by real-time RT-PCR, was highly increased. (**b**) Human CF were stimulated for 24 h with 1 ng/ml PolyIC added to the medium to stimulate membrane-bound receptors, such as TLR3, or with 1 ng/ml of intracellularly transfected PolyIC to stimulate cytosolic receptors, such as MDA5 and RIG-I. IL-6, IL-8, and TNF-α, but not IL-1β, measured at the protein level in cell-free supernatants, were significantly higher after cytosolic receptors stimulation (transfected PolyIC) than after stimulation of membrane-bound receptor (PolyIC). (**c**-**d**) Knockdown of TLR3 with siRNA in human CF stimulated for 2, 4, and 8 h with 1 ng/ml of intracellularly transfected PolyIC for MDA5 and RIG-I stimulation. SiMock was used as a control. Real-time RT-PCR used to measure transcription of *IL-1β*, *IL-6*, *IL-8*, and *TNF-α* demonstrated that TLR3 partially contributed to cytosolic production of pro-inflammatory cytokines (**c**) but did not support direct expression of *MDA5* and *RIG-I* (**d**). Means ± s.d. and values measured from one out of two independent experiments performed in duplicates are shown. Unpaired two-tailed Student t test (b), **p < 0.01 for PolyIC vs. Transf. PolyIC; two-way ANOVA with Bonferroni post hoc testing (c), **p < 0.01 for siMock vs. siTLR3

TLR3 is not the only pattern recognition receptor (PRR) that is activated upon PolyIC stimulation. Other PRRs, namely the cytosolic RLRs, such as MDA5 and RIG-I, are also activated by PolyIC, and more precisely in the cellular cytosol [32]. Since PolyIC have to pass through the cell membrane to reach the cytosol and to activate MDA5 and RIG-I, PolyIC was transfected intracellularly into CF by lipofection. Expression of pro-inflammatory cytokines evaluated at the RNA level showed that expression of *IL-1β*, *IL-6*, *IL-8*, and *TNF-α* in PolyIC-transfected CF rose in parallel with the increased expression of MDA5 and RIG-I, but not with the expression of TLR3 (Fig. 4a). Remarkably, when pro-inflammatory cytokines measured at the protein level in supernatants of PolyIC-stimulated CF without transfection, whereby membrane-bound TLR3 was activated, were compared to PolyIC-transfected CF, whereby cytosolic MDA5 and RIG-I were activated, the latter produced significantly higher amounts of IL-6, IL-8 and TNF-α (Fig. 4b). To exclude a potential involvement of TLR3 after cytosolic PolyIC stimulation in human CF, TLR3 was first knocked down by siTLR3, and then CF were transfected with PolyIC for cytosolic stimulation. Interestingly, TLR3 knockdown significantly reduced the expression of *IL-1β*, *IL-6*, and *IL-8* (Fig. 4c). Conversely, the expression of *TNF-α* was slightly increased after TLR3 knockdown (Fig. 4c). The expression of MDA5 and RIG-I in TLR3 knockdown CF was not affected, demonstrating that a potential mechanism that counter-balanced the absence of TLR3 was not necessary (Fig. 4d). It is to notice that the kinetics of cytokine expression was delayed, probably because the CF underwent two consecutive transfections, namely the first with siRNA and the second with PolyIC. It is likely that the first transfection already induced a slight cellular activation that led to CF exhaustion and consequently to generally delayed expression of pro-inflammatory cytokines.

### Pro-Inflammatory Cytokines Produced in Human CF after Intracellular PolyIC Stimulation are Dependent on RLRs Receptors

**Fig. 5:**
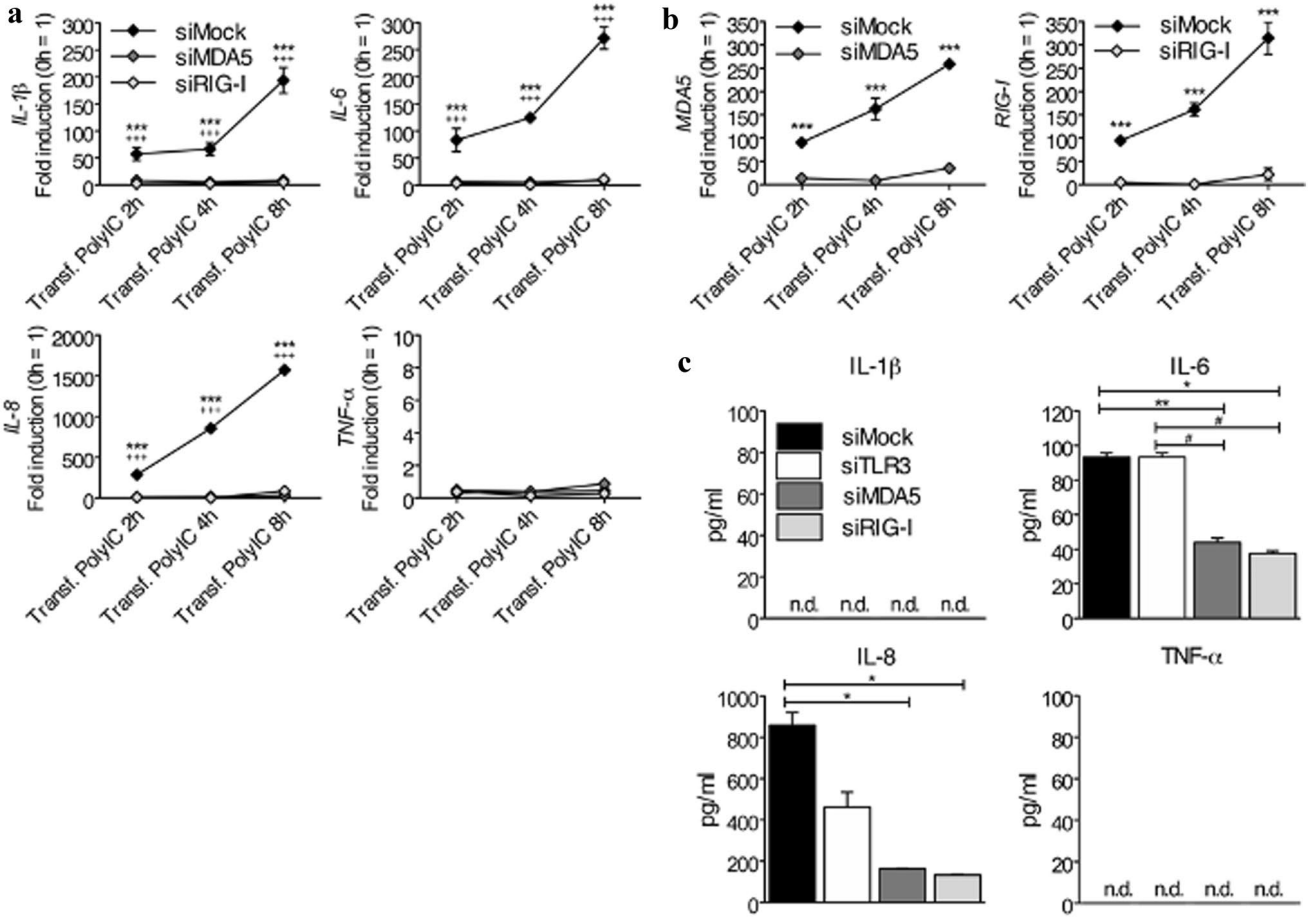
MDA5 and RIG-I regulate pro-inflammatory cytokines in human CF after intracellular PolyIC stimulation. (**a**-**b**) Knockdown of MDA5 and RIG-I with siRNA in human CF stimulated for 2, 4, and 8 h with 1 ng/ml of intracellularly transfected PolyIC. SiMock was used as a control. Transcription of *IL-1β*, *IL-6*, *IL-8*, but not of *TNF-α*, after stimulation with cytosolic PolyIC, was MDA5- and RIG-I-dependent (**a**). *MDA5* and *RIG-I* knock-down efficiency was also determined by real-time RT-PCR (**b**). (**c**) Knockdown of TLR3, MDA5, and RIG-I with siRNA in human CF stimulated for 24 h with 1 ng/ml of intracellularly transfected PolyIC. SiMock was used as a control. IL-6 and IL-8, but not IL-1β and TNF-α, all measured at the protein level in cell-free supernatants, showed reduced levels after siRNA-induced knockdown. n.d., not detected. Means ± s.d. and values measured from one out of two to three independent experiments performed in duplicates are shown. Two-way ANOVA with Bonferroni post hoc testing, ***p < 0.001 for siMock vs. siMDA5, ^+++^*p* < 0.001 for siMock vs. siRIG-I (a); ***p < 0.001 for siMock vs. siMDA5 (left panel) or siMock vs. siRIG-I (right panel) (b); *p < 0.05, **p < 0.01 for siMock vs. siMDA5 and siMock vs. siRIG-I, ^#^*p* < 0.05 for siTLR3 vs. siMDA5 and siTLR3 and siRIG-I (c)

To confirm the RLRs-dependent regulation of pro-inflammatory cytokines in human CF upon stimulation with transfected PolyIC, MDA5 and RIG-I were knocked down with specific siRNA (siMDA5 and siRIG-I, respectively) and then stimulated with transfected PolyIC. As shown in Fig. 5a, both MDA5 and RIG-I knockdown completely abrogated the expression of *IL-1β*, *IL-6*, and *IL-8*, while *TNF-α* was low after all transfections. As already observed in Fig. 4c, the kinetics of cytokine expression was slower than in CF which underwent just one transfection. Almost complete depletion of MDA5 and RIG-I RNA expression in siMDA5- and siRIG-I-transfected CF demonstrated the high efficiency of the siRNA used in these experiments (Fig. 5b). At the protein level, however, besides TNF-α, also IL-1β was not detectable in supernatants of CF, while IL-6 and IL-8 production was significantly lower after siMDA5- and siRIG-I-knockdown than after siTLR3- and siMock-knockdown (Fig. 5c). In addition, IL-6 measured in supernatants collected from siMock- and siTLR3-transfected CF were comparable, suggesting that MDA5 and RIG-I, but not TLR3, control the cytosolic signalling pathway triggered by PolyIC in human CF.

### Human CF Express IFN-β upon Stimulation with PolyIC

**Fig. 6:**
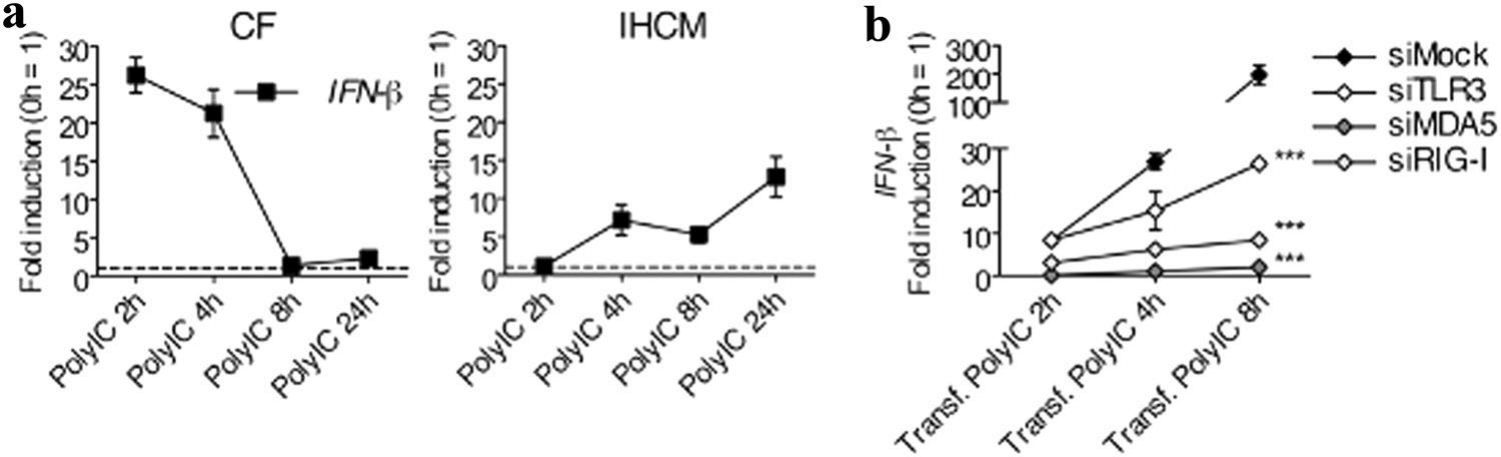
Human CF, and partially the IHCM, induce *IFN-β* expression after intracellular PolyIC stimulation. (**a**) Human CF and IHCM were stimulated for 2, 4, 8, and 24 h with 1 ng/ml of intracellularly transfected PolyIC. Transcription of *IFN-β*, determined by real-time RT-PCR, was increased in both human CF and in the IHCM. (**b**) Knockdown of TLR3, MDA5, and RIG-I with siRNA in human CF stimulated for 2, 4, and 8 h with 1 ng/ml of intracellularly transfected PolyIC. SiMock was used as a control. Transcription of *IFN-β*, which was determined by real-time RT-PCR, was MDA5-, RIG-I-, und TLR3-dependent. Means ± s.d. and values measured from one out of three independent experiments performed in duplicates are shown. Two-way ANOVA with Bonferroni post hoc testing, ***p < 0.001 for siMock vs. siTLR3, siMock vs. siMDA5, and siMock vs. siRIG-I (b)

Anti-viral responses are induced by different cells that produce type I IFNs, which include IFN-α and IFN-β [42]. Since PolyIC is a strong inducer of anti-viral type I IFNs [32], human CF and the IHCM were first stimulated with untransfected PolyIC to induce *IFN-β* expression through TLR3. The kinetics of *IFN-β* expression was different between CF and the IHCM. Indeed, stimulated CF induced a fast response in the first 4 h of stimulation, while in the IHCM, *IFN-β* expression increased gradually but never reached the amplitude of the response induced by CF (Fig. 6a). It is known that MDA5 and RIG-I promote IFN-β production in leukocytes [43]. To verify whether cytosolic RLRs also induce *IFN-β* expression in human cardiac cells, PolyIC was transfected in CF. In addition, to determine the specific cytosolic pathway that regulates *IFN-β* expression in CF, TLR3, MDA5, and RIG-I were knocked down with specific siRNA. In siMock-treated CF, stimulation with transfected PolyIC induced a strong IFN-β response, which was reduced by about ten times after TLR3 knockdown, and even more after MDA5 and RIG-I knockdown (Fig. 6b). These data suggest that not only MDA5 and RIG-I, but also TLR3, control *IFN-β* expression in human CF upon PolyIC stimulation.

## DISCUSSION

In this study, we showed that human CF and the IHCM differently reacted to TLRs stimuli. Only human CF produced pro-inflammatory cytokines, while the IHCM was mostly unresponsive. Upon specific stimulation of TLR4, human CF consistently induced IL-6 and IL-8, while TLR2 was not expressed on either CF or the IHCM. Upon stimulation of TLR3 with PolyIC, human CF expressed most of the investigated pro-inflammatory cytokines. The remarkable increase of pro-inflammatory cytokines after PolyIC transfection into the cytosol of human CF demonstrated that the cytosolic RLRs receptors MDA5 and RIG-I were activated in human CF and contributed to induce inflammation. Furthermore, IFN-β was upregulated in human CF through activation of TLR3, MDA5, and RIG-I.

Some of our findings are comparable with results obtained using human and mouse cardiac cell lines. Indeed, in human CF, TLR4 stimulation with LPS increased the production of IL-1β, IL-2, IL-6, IL-8, TNF-α, and IFN-γ, leading to impaired physiological CF function [44]. Similarly, in primary mouse CF, but also in primary mouse CM, TLR4 stimulation with LPS promoted IL-6 and TNF-α production [24, 28]. However, although the IHCM is an immortalized cell line and we do not know if the immortalization process affected TLRs expression and the potential to express cytokines, our results in the IHCM were in contrast with published data in mouse and rat CM. In fact, in mice and rats with induced MI, TLR4 has been detected in CM [45]. The same rat CM produced higher concentrations of IL-6 and TNF-α after LPS stimulation when compared to CM of healthy mice [45]. The mouse H9C2 CM cell line and primary rat CM stimulated with the heat shock protein 60 (HSP60) produced several pro-inflammatory cytokines in a TLR2- and TLR4-dependent manner [46]. In our study, the IHCM was unable to mount TLRs-dependent inflammatory responses, but rather slightly expressed IFN-β after RLRs stimulation. Furthermore, we found that TLR2, TLR3, and TLR4 were not expressed in the IHCM, while in mouse and rat CM, their expression has been described in several studies [27, 47]. These data indicate that the IHCM used in our study cannot respond to TLRs stimuli.

Diversities in activation of the transcription factor NF-kB have been found between mouse CF and CM. Studies performed with mouse primary heart cells demonstrated that the nuclear translocation and phosphorylation of NF-kB was different between CF and CM [37, 40]. Depending on the stimulating agent used, CF or CM expressed different levels of pro-inflammatory cytokines [37, 40]. In terms of anti-viral type I IFNs production, mouse CM showed higher concentrations of basal type I IFN than CF [48]. On the contrary, mouse CF infected with the cardiotropic Coxsackievirus B3 (CVB3) expressed higher values of IFN-β than CVB3-infected CM [49]. Here we proved that both human CF and the IHCM expressed IFN-β after TLR3 stimulation. Furthermore, cytosolic stimulation was dependent on TLR3, MDA5, and RIG-I, indicating that human CF can produce IFN-β without the co-operation of cells of the immune system. This is important during viral infections with cardiotropic viruses, such as the CVB3. Transgenic mice over-expressing MDA5 in cardiac tissue showed increased levels of IFN-β in the heart, which conferred higher protection against virus-induced myocarditis [50]. In humans, elimination of the virus from the heart was successfully achieved in patients suffering from acute enteroviral- or adenoviral-induced myocarditis after treating them with 18 × 10^6^ IU/week IFN-β for 24 weeks [51]. Improved heart function and reduced inflammatory cells in the heart after treatment with IFN-β, which lowered the risk of progression to dilated cardiomyopathy, where also observed in an additional study with Parvovirus B19-infected patients [51, 52]. In addition, IFN-β has been proposed as treatment option in patients with severe acute respiratory syndrome Coronavirus-2 (SARS-CoV2)-associated myocarditis [53]. Collectively, it is likely that CF, by initiating an anti-viral response, co-operate with heart-resident macrophages and promote the recruitment of other immune cells, such as neutrophils, monocytes, and natural killer cells, which support a quick innate immune response to reduce the detrimental effects of the invading pathogens. This could explain why TLRs and RLRs are expressed in cardiac cells.

Pro-inflammatory cytokines play relevant functions in heart inflammation. Evidence that IL-1β, IL-6, and TNF-α exacerbate heart function in patients with MI led to the hypothesis that inhibiting their function would ameliorate patients’ clinical outcome. Indeed, treatment with canakinumab, which is a specific monoclonal antibody targeting IL-1β, reduced the risk of cardiovascular events in patients with previous MI and high CRP [54]. The anti-IL-6 receptor antibody Tocilizumab attenuated inflammation in MI patients and reduced troponin T release [55]. However, other treatments to reduce IL-1β with other compounds, such as Anakinra, or the use of anti-TNF-α treatments, such as Etanercept, did not show the expected benefits [56]. On the contrary, adverse effects of the anti-TNF-α antibody Infliximab have been described in patients with heart failure (HF) [57]. In general, the treatment of MI patients with anti-IL-1β, anti-IL-6, and anti-TNF-α antibodies reduced the levels of C-reactive protein (CRP) [56]. In patients with myocarditis, increased amounts of IL-1β and IL-6 correlated with higher levels of pathogenic T helper 17 cells [58]. According to recent data, human respiratory viruses, such as the SARS-CoV-2, can manifest with heart inflammation accompanied by hyperinflammatory conditions, such as cytokine-release syndrome (CRS). Increases in IL-6 have been observed in patients with advanced disease [59]. Pro-inflammatory cytokines contributed to this myocarditis-like syndrome involving acute myocardial injury [60]. Therefore, although reduction of pro-inflammatory cytokines could limit heart diseases, it is crucial to find a balance between their damaging and their protecting function. In the present study, human CF produced IL-6, IL-8, and IFN-β, but it is likely that their physiological amounts are not so high to induce heart inflammation. These cytokines are necessary for a first unspecific but crucial protection against pathogens. It is also true that many heart diseases are not the result of an infection with a pathogenic agent, hence cytokines produced by human CF can further deteriorate heart function and their purpose cannot be considered protective. In addition, heart-resident as well as heart-infiltrating immune cells recruited to the heart by cytokines and chemokines produced by CF can mount uncontrolled responses that ultimately damage the heart tissue, leading to dilated cardiomyopathy and HF. Additional studies will be necessary to figure out the physiological function of cytokines produced by CF.

Taken together, we demonstrate that human CF, but not the IHCM, produce the pro-inflammatory cytokines IL-6 and IL-8 upon TLR3, TLR4, MDA5, and RIG-I stimulation. Human CF can produce IFN-β. These findings will help to further understand how single heart cell populations, heart-resident macrophages and heart-infiltrating immune cells react to inflammatory stimuli and how they interact to each other, to ultimately support the development of new strategies aiming to limit excessive and detrimental activation of the immune system in the heart.

## Data Availability

The datasets generated during and/or analyzed during the current study are available from the corresponding author on reasonable request.
